# Synthesis of Bottlebrush Polymers with Spontaneous Self-Assembly for Dielectric Generators

**DOI:** 10.1021/acsapm.3c03053

**Published:** 2024-04-22

**Authors:** Yeerlan Adeli, Thulasinath Raman Venkatesan, Raffaele Mezzenga, Frank A. Nüesch, Dorina M. Opris

**Affiliations:** † Laboratory for Functional Polymers, Swiss Federal Laboratories for Materials Science and Technology Empa, Ueberlandstr. 129, CH-8600 Dübendorf, Switzerland; ‡ Institute of Chemical Sciences and Engineering, Ecole Polytechnique Federale de Lausanne, EPFL, Station 6, CH-1015 Lausanne, Switzerland; § Department of Health Sciences and Technology, ETH Zürich, Laboratory of Food and Soft Materials, Schmelzbergstrasse 9, 8092 Zürich, Switzerland; ∥ Department of Materials, ETH Zurich, Vladimir-Prelog-Weg 5, 8093 Zurich, Switzerland

**Keywords:** dielectrics, bottlebrush polymers, electrically responsive elastomers, ROMP, self-segregated copolymers

## Abstract

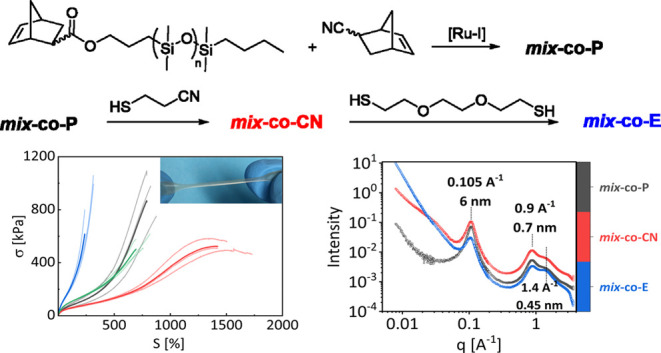

Cross-linked bottlebrush polymers received significant attention as dielectrics in transducers due to their unique softness and strain stiffening caused by their structure. Despite some progress, there is still a great challenge in increasing their dielectric permittivity beyond 3.5 and cross-linking them to defect-free ultrathin films efficiently under ambient conditions. Here, we report the synthesis of bottlebrush copolymers based on ring-opening metathesis polymerization (ROMP) starting from a 5-norbornene-2-carbonitrile and a norbornene modified with a poly(dimethylsiloxane) (PDMS) chain as a macromonomer. The resulting copolymer was subjected to a postpolymerization modification, whereby the double bonds were used both for functionalization with thiopropionitrile and subsequent cross-linking via a thiol–ene reaction. The solutions of both bottlebrush copolymers formed free-standing elastic films by simple casting. DMA and broadband impedance spectroscopy revealed two glass transition temperatures uncommon for a random copolymer. The self-segregation of the nonpolar PDMS chains and the polynorbornane backbone is responsible for this and is supported by the interfacial polarization observed in broadband impedance spectroscopy and the scattering peaks observed in small-angle X-ray scattering (SAXS). Additionally, the modified bottlebrush copolymer was cross-linked to an elastomer that exhibits increased dielectric permittivity and good mechanical properties with significant strain stiffening, an attractive property of dielectric elastomer generators. It has a relative permittivity of 5.24, strain at break of 290%, elastic modulus at 10% strain of 380 kPa, a breakdown field of 62 V μm^–1^, and a small actuation of 5% at high electric fields of 48.5 V μm^–1^. All of these characteristics are attractive for dielectric elastomer generator applications. The current work is a milestone in designing functional elastomers based on bottlebrush polymers for transducer applications.

## 1 Introduction

Bottlebrush polymers are composed of repeat units bearing long side chains.^1^ The side chains stretch the polymer backbone and prevent it from coiling and entangling because of steric hindrance and entropic repulsion. Therefore, unlike elastomers made by cross-linking linear polymers with many entanglements, bottlebrush polymers entangle less.^2−4^ Therefore, cross-linked bottlebrush polymeric materials exhibit unique mechanical properties, such as super softness at small strains due to the brush’s plasticizing effect and stiffness at large strains due to the backbone being stretched toward its full contour length.^5^ Strain stiffening is desired in many applications such as dielectric elastomer actuators and generators, which are thin elastic capacitors. Because materials with an increased elastic modulus exhibit higher dielectric breakdown strength, strain stiffening of an elastomer can prevent premature breakdown^6,7^ and improve devices’ performance. For example, dielectric generators can be operated at higher voltages and thus harvest more energy per cycle, and dielectric actuators may not experience electromechanical instability (EMI).^8^ As the voltage increases, soft elastomer films undergo thinning, increasing the local electric field and leading to further thinning of the film. This kind of positive feedback between the thickness of a film and the local electric field often causes dielectric breakdown. As a result, the materials fail before reaching their full potential. Strain stiffening in elastomers disrupts the positive feedback between the thinning of the membrane under increasing electric fields and, thus, can prevent EMI from happening.

Ring-opening metathesis polymerization (ROMP)^9^ has been used to prepare bottlebrush polymers^10−17^ because it is compatible with various functional groups^18−21^ and has a fast living polymerization rate.^22−24^ In 2018, Beers et al. synthesized a bottlebrush polymer elastomer via ROMP. They used a norbornene macromonomer functionalized with a poly(*n*-butyl acrylate) chain and a bifunctional norbornene macromonomer as a cross-linker.^25^ In 2020, Bates et al. reported a bottlebrush polymer with poly(dimethylsiloxane) (PDMS) side chains prepared by ROMP, which was subsequently cross-linked by a PDMS containing two benzophenone end groups.^26^ The cross-linking reaction was conducted in a special mold to avoid oxygen inhibiting the free-radical-based reaction. Therefore, constrained by the indispensable mold, the resulting membrane exhibited a substantial thickness of 0.4 mm. Recently, Cushman et al. reported a bottlebrush polymer prepared starting from a macromonomer consisting of a PDMS chain with one norbornene end group and a PDMS cross-linker with two norbornene end groups.^27^ The cross-linking and polymerization were accomplished in situ inside a glovebox. Despite the increasing number of publications on elastomers based on bottlebrush polymers, efficient ways to produce thin films with no defects under air are scarce. Furthermore, the bottlebrush polymer prepared had the desired mechanical properties, but none exhibited a dielectric permittivity (ε′) above 3.5.

Soft and stretchable dielectric elastomers with high dielectric permittivity are required in many applications, including transducers,^28−30^ soft robots,^31−35^ energy harvesting,^36,37^ energy storage,^38^ and printable and stretchable electronics.^39^ For example, in energy harvesting devices, high ε′ and high strain at break can enhance the maximum energy harvested, while a small elastic modulus can be helpful to couple material’s response with various intensities and frequencies of input excitations.^40^ Besides, since materials with high dielectric permittivity and good mechanical properties are also used as electrolytes, they are highly demanded in ionic actuators, solid-state Li-ion batteries, and electrochromic devices.^38^

Recently, we demonstrated a series of ROMP-based bottlebrush polymers with PDMS brushes and could cross-link them in the air to defect-less thin films with good mechanical properties.^41^ However, the employed nonpolar macromonomer resulted in elastomers with a relatively low dielectric permittivity. Here, we want to explore the possibility of achieving soft and elastic materials by a ROMP starting from a polar norbornene monomer and a macromonomer consisting of a norbornene modified with a PDMS chain. The formed copolymer was expected to exhibit increased dielectric permittivity and an enhanced strain stiffening effect since the polar small monomer reduced the density of brushes.^42^ Additionally, the C–C double bonds in the polymer backbone offer the possibility of postpolymerization modification via thiol–ene addition with polar thiols to increase the dielectric permittivity and cross-linking with multifunctional thiols to tune the mechanical properties.

## 2 Experimental Section

### 2.1 Materials

PDMS, monohydride terminated (AB250915, viscosity 5–9 cSt.) (*M_n_* = 1136 g/mol determined by ^1^H NMR end-group analysis), was purchased from ABCR. Karstedt’s catalyst (platinum(0)-1,3-divinyl-1,1,3,3-tetramethyldisiloxane complex solution in xylene, Pt ≈ 2%), allyl alcohol, 4-(dimethylamino)pyridine (DMAP), *N*,*N*′-diisopropylcarbodiimid (DIC, 18.4 mL, 122.4 mmol, 3.4 equiv), 5-norbornene-2-carboxylic acid (mixture of *endo* and *exo*, predominantly *endo*), 5-norbornene-2-carbonitrile, first-generation Grubbs’ catalyst, 2,2-dimethoxy-2-phenylacetophenone (DMPA), and 2,2′-(ethylenedioxy)diethanethiol (**CL**) were purchased from Sigma-Aldrich. Poly(vinyl alcohol) (PVA, R&G-PVA-Folientrennmittel) was purchased from Suter-Kunststoff AG. Methanol (MeOH), dichloromethane (DCM), ethyl acetate (EA), toluene (Tol), tetrahydrofuran (THF), and heptane were purchased from VWR. All chemicals were of reagent grade and used without purification; only toluene was dried over sodium using benzophenone as an indicator and DCM over calcium hydride and distilled before use.

### 2.2 Characterization

More details about the characterization and equipment used can be found in the Supporting Information.

The macromonomer ***mix*****-M** was synthesized according to our previous publication.^41^

### 2.3 Synthesis of Bottlebrush Polymer ***mix*****-*****co*****-P**

The macromonomer, ***mix*****-M** (20 g, 21.3 mmol, 2 equiv), and the *co*-monomer, 5-norbornene-2-carbonitrile (3.8 g, 31.9 mmol, 3 equiv), were put in a flask and backfilled with Ar, followed by the addition of dry DCM (250 mL). Then, the mixture was degassed 3 times, and to the degassed mixture, the first-generation Grubbs’ catalyst (23 mg, 26.6 μmol, 0.00625 equiv) was added. After the mixture was heated to reflux at 45 °C for 32 h, ethyl vinyl ether (2 mL) was added to quench the reaction. Next, the product was purified by precipitation from DCM with MeOH. After purification, the bottlebrush polymer (***mix*****-*****co*****-P**) was kept as a solution of toluene at a concentration of 200–500 mg/mL.

To prepare thin films, the solution was placed on a Teflon substrate and cast by a doctor blade. The blade thickness was adjusted to 400–500 μm, for a concentration of polymer of about 200–500 mg/mL. After blade casting the solution mixture, the solvent was let to evaporate for 1 h at room temperature and then put in a vacuum oven at 60 °C overnight to remove the residual solvents.

### 2.4 Synthesis of ***mix*****-*****co*****-CN**

The mixture of bottlebrush polymer, ***mix*****-*****co*****-P** (10 g, ∼10.0 mmol, 1 equiv), 3-mercapto propionitrile (1.776 g, 20 mmol, 2 equiv), and DMPA (20 mg, 0.1 mmol, 1 mol %) was put in a flask and degassed and backfilled with Ar 3 times, followed by ultraviolet (UV) irradiation for 20 min. Next, the product was purified by precipitation from DCM to MeOH. After the purification, the bottlebrush polymer (***mix*****-*****co*****-CN**) was kept as a toluene solution at a 200–500 mg/mL concentration. The preparation of thin films ***mix*****-*****co*****-CN** is the same as that of ***mix*****-*****co*****-P**.

### 2.5 Preparation of Material ***mix*****-*****co*****-E**

A solution of **CL** (10%, v/v) in toluene was prepared. A mixture of the corresponding amount of bottlebrush polymer solution, **CL**, and 2,2-dimethoxy-2-phenylacetophenone (DMPA, 2 wt % to bottlebrush polymer) was put in a vial and mixed well by centrifugation. Next, the mixture was put on a Teflon substrate and cast with a doctor blade with a certain thickness. After blade coating the solution mixture, it was irradiated for 5 min with UV to cross-link the films. A Hönle UVA HAND 250 GS UV lamp was used 12 cm from the substrate as the UV source. Before the characterization, all of the materials were put in a vacuum oven at 60 °C overnight to remove the residual solvents.

## 3 Results and Discussion

**Scheme 1:**
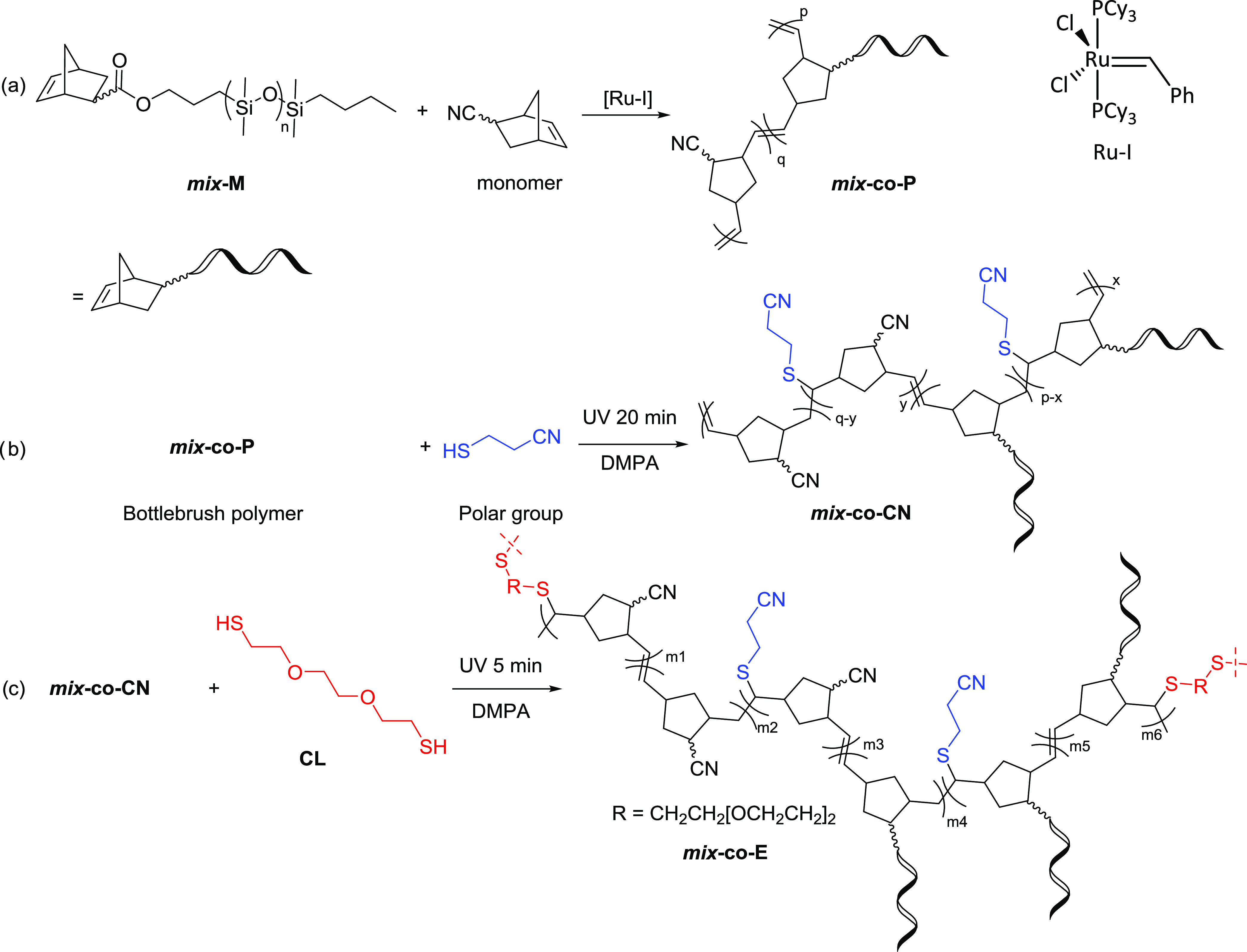
Synthetic Path to Bottlebrush Elastomers via ROMP of Macromonomer ***mix*****-M** and Monomer 5-Norbornene-2-carbonitrile (a) Followed by polar group functionalization by the Thiol–Ene reaction of 3-mercapto propionitrile with double bonds from ***mix*****-*****co*****-P** Backbone to give ***mix*****-*****co*****-CN** (b), and subsequent cross-linking by the Thiol–Ene reaction of 2,2′-(ethylenedioxy)diethanethiol (CL) with double bonds from ***mix*****-*****co*****-CN** backbone to form elastomers ***mix*****-*****co*****-E** (c).

The synthetic strategy for chemically cross-linked bottlebrush polymers is illustrated in Scheme 1. A mixture of *endo* and *exo* 5-norbornene-2-carbonitrile was chosen as a polar monomer to increase the dielectric permittivity and reduce the brush density on the polymer backbone, while the second monomer was a mixture of endo and exo norbornene modified with a commercially available short PDMS chain with a viscosity of 5–9 cSt. and *M_n_* = 1136 g/mol as determined by ^1^H NMR end-group analysis. The mechanical properties of bottlebrush polymeric elastomers with various brush densities are influenced by many factors like side chain length, degree of polymerization, the chemical structure of the bottlebrush polymer, etc., and thus, are difficult to predict.^42,43^ It has been shown that strain stiffening is more pronounced for materials obtained by cross-linking bottlebrush polymers with a reduced density of brushes on the backbone; however, the price is an increased elastic modulus.^43^ Additionally, it was observed that the mechanical properties of the cross-linked bottlebrush polymer material did not change so much when the density of brushes was 50%. Therefore, the molar ratio between the macromonomer and 5-norbornene-2-carbonitrile was chosen to be 4:6. This will allow us to achieve bottlebrush polymers with increased dielectric permittivity and reduced brush density so that eventually, an elastomer soft at small strains, which stiffens with the strain, is achieved.

A mixture of *endo* and *exo* monofunctional norbornene end-terminated PDMS macromonomers (***mix*****-M**) was first prepared according to the literature by an esterification reaction between a mixture of *exo* and *endo* isomers of 5-norbornene-2-carboxylic acid and a monohydroxyl terminated PDMS with an average of 14 dimethylsiloxy repeat units (Scheme 1).^41^ Though the macromonomer prepared from *exo* 5-norbornene-2-carboxylic acid possesses a higher reactivity and is favored in ROMP, it is more expensive than its counterpart with mixed configurations.

**Figure 1:**
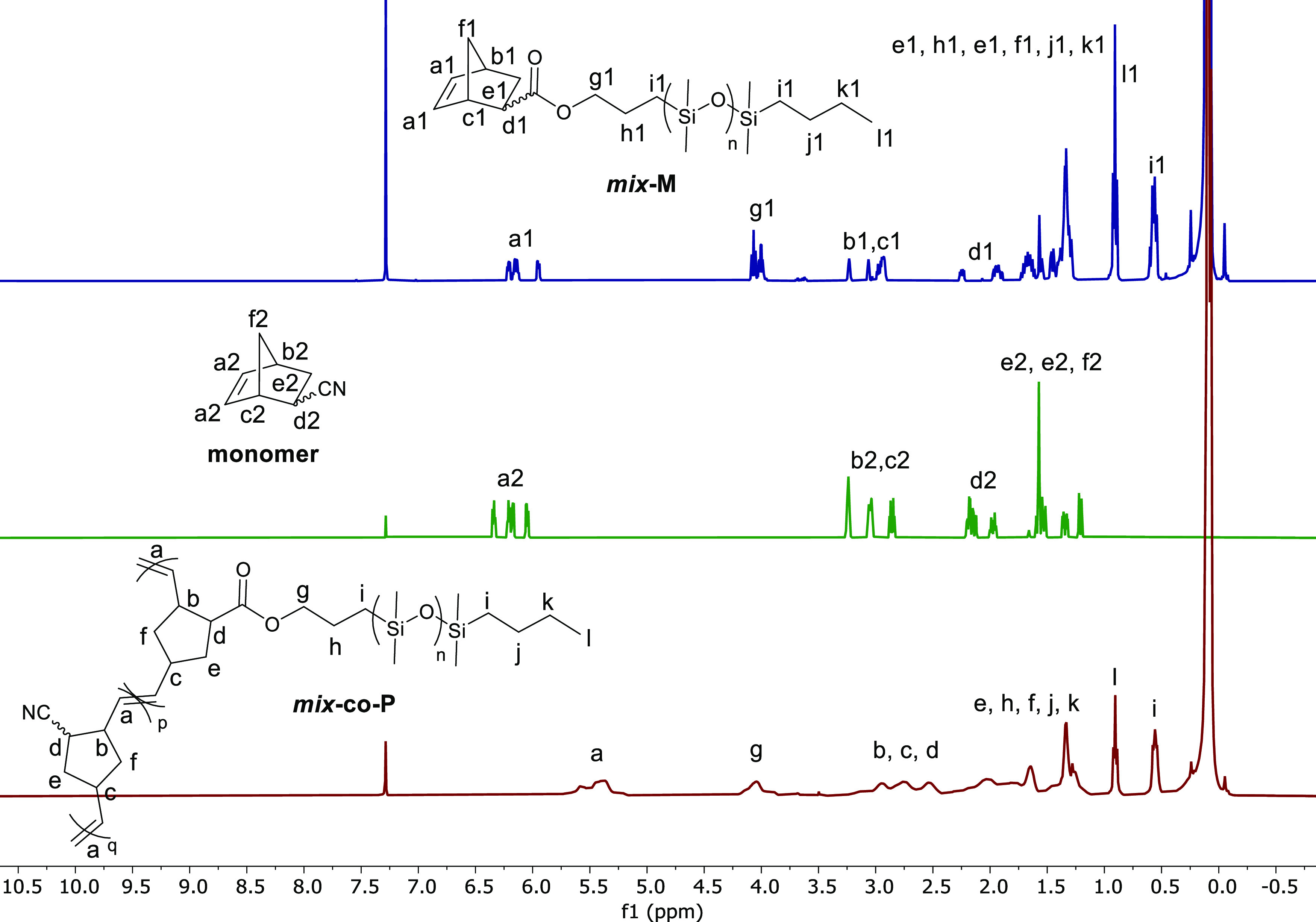
^1^H NMR spectra of macromonomer ***mix*****-M** (top), 5-norbornene-2-carbonitrile (middle), and polymer******mix*****-*****co*****-*********P**** (bottom).

ROMP of ***mix*****-M** and 5-norbornene-2-carbonitrile with a molar ratio of (2:3) using first-generation Grubbs’ catalysis as an initiator allowed the formation of a bottlebrush polymer ***mix*****-*****co*****-P** (Scheme 1a). The shifting of starting materials’ vinylene group signals from 6.0 to 6.5 of the monomer to 5.1 to 5.6 ppm in the polymer in ^1^H NMR spectra marked the formation of product ***mix*****-*****co*****-P** and accomplishment of the *co*-polymerization (Figure 1). Furthermore, the molar ratio between the two repeating units of bottlebrush polymer ***mix*****-*****co*****-P** was determined by ^1^H NMR spectroscopy by using the signal of the methyl end group from the brush of the macromonomer at 0.9 ppm and the one on the vinylene groups between 5.1–5.6 ppm (*p*/*q* = 2:2.7), resembling the initial ratio before the reaction (Figures S1 and S2).

**Figure 2:**
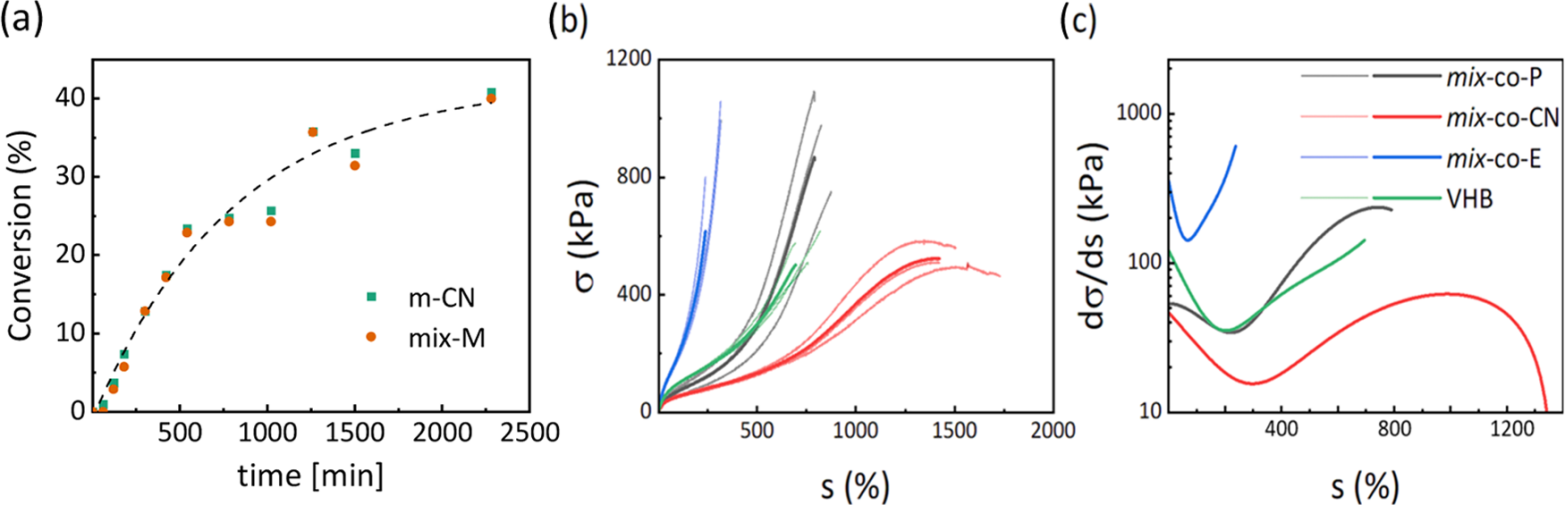
Conversion of ***mix***-**M** and 5-norbornene-2-carbonitrile over time. The dashed line is a guide to the eye. (a) Engineering stress–strain curves for materials ***mix*****-*****co*****-P**, ***mix*****-*****co*****-CN**, ***mix*****-*****co*****-E**, and **VHB**. The light curves are the results from 3 independent measurements of the same material, while the bold curves are the averaged curves of the same material, using Origin software, whereby the smaller strain at break is shown. For the average strain at break, see Table 1 (b) and the curves of dσ/ds at different strains (c).

Additionally, ^1^H NMR spectra of samples taken from the reaction mixture at different reaction times show that the proportion of the vinylene hydrogens of the different monomers remained unchanged throughout the polymerization, suggesting a random incorporation of the monomers into the polymer chain (Figures 2a, S2 and S3 and Table S1). Moreover, the molar mass of the polymer increases with the reaction time (Figure S4). Although macromonomer ***mix*****-M** had a much larger molar mass than that of 5-norbornene-2-carbonitrile, they exhibited similar reactivity. However, the overall conversion after about 40 h was only 40%, probably due to the interference of nitrile groups with the Ru initiator. While the literature suggests that the third-generation Grubbs’ initiator gives higher conversion in homopolymerization of 5-norbornene-2-carbonitrile, the first-generation Grubbs’ initiator is much cheaper.^44^ The purification of the polymer from the unreacted monomers was by precipitation.

Polymers of high molar mass are indispensable for good elastic properties.^45^ Therefore, the bottlebrush polymer ***mix*****-*****co*****-P** was characterized by gel permeation chromatography (GPC) to determine its molar mass and polydispersity index (PDI), which gave *M_n_* = 136.4 kDa and PDI = 1.89 (Figure S5 and Table S2). However, these values should be taken with precaution due to the compact structure of bottlebrush polymers and the great difference between the GPC standard structure and the polymer analyzed.

After the solvent was removed, we observed that the bottlebrush polymer exhibited interesting elastic properties even when not chemically cross-linked. Thus, thin films were made by doctor blading a solution of bottlebrush polymer on a Teflon substrate and letting the solvent evaporate. It should be noted that the homopolymer synthesized starting from ***mix***-**M** previously reported by our group behaved differently, e.g., the bottlebrush polymer was a viscous liquid that did not cross-link after solvent evaporation.^41^ Therefore, it can be concluded that the presence of the second polar repeat unit in ***mix*****-*****co*****-P** is responsible for the film cohesion, as will be discussed later.^46^ PDMS easily undergoes phase segregation even when mixed with polysiloxanes that carry different functional groups. Therefore, it is very likely that the polysiloxane brushes and the polynorbornene backbone phase segregate, whereby self-segregation of the polynorbornene backbone is facilitated by the dipole–dipole interaction of the repeat units that carry a nitrile group.

Additionally, the double bonds from the polynorbornene backbone offered us the possibility of postpolymerization modification by thiol–ene reaction with 3-mercapto propionitrile to enhance the dielectric permittivity and possibly reduce the elastic modulus. The resulting polymer was named ***mix*****-*****co*****-CN** (Scheme 1b).^41^ The result from GPC showed the *M_n_* to be 209.3 kDa and the PDI to be 2.16 (Figure S5 and Table S2). According to the ^1^H NMR spectroscopy analysis, the double bond conversion reached 9% (Figure S6). Also this modified bottlebrush polymer forms free-standing films when dried. It should be noted that films of ***mix*****-*****co*****-P** and ***mix*****-*****co*****-CN** can be reprocessed by dissolving in suitable solvents such as THF. The unreacted double bonds of ***mix*****-*****co*****-CN** were further used for cross-lining into thin films using 2,2′-(ethylenedioxy)diethanethiol to give ***mix*****-*****co*****-E**.

The films were subjected to tensile tests on samples in the shape of a dumbbell (Figure 2b). An average curve in bold lines was calculated from three independent measurements (light curves) for each material. Furthermore, we also tested **VHB**, a double-sided acrylate adhesive tape produced by the company 3 M known for its high strength, as a model material for comparison as it has been intensively explored as a dielectric in actuators and generators. We calculated the elastic modulus and average strain at break of different materials presented in Figure 2b (Table 1). All materials showed a rather large strain at break, with some reaching values as high as 1553%. Both materials ***mix*****-*****co*****-P** and ***mix*****-*****co*****-CN** had a strain at break larger than that of **VHB**. The high *Y*_10%_ elastic modulus of all materials should be noted, which is likely due to the dipolar interaction since this behavior was absent in the homo bottlebrush polymer without polar blocks previously reported by our group.^41^ Although they showed a relatively large elastic modulus at small strains, it dropped to lower levels above a certain strain and increased again at higher strain levels.

**Table 1:** Mechanical Properties of ****mix******-*****co*****-P**, ****mix*****-*****co******-CN**, ****mix*****-*****co******-E**, and **VHB**

entry	*Y*_10%_ [kPa]^a^	*s*_0_ [%]^b^	*Y*_min_ [kPa]^c^	*Y*_max_ [kPa]^d^	*Y*_max_/*Y*_min_	*s*_max_ [%]^e^
***mix-co*****-P**	166 ± 28	220	34 ± 9	199 ± 53	5.9	830 ± 43
***mix*****-*****co*****-CN**	151 ± 8	300	17 ± 2	68 ± 6^f^	3.9	1553 ± 159
***mix*****-*****co*****-E**	381 ± 27	65	147 ± 17	902 ± 84	6.1	289 ± 45
**VHB**	230 ± 3	200	38 ± 3	103 ± 26	2.7	757 ± 62

a Elastic modulus at 10% strain.

b The strain where the materials start stiffening.

c Elastic modulus at *s*_0_.

d Elastic modulus at the strain of break.

e Average strain at break of three samples.

f Elastic modulus at 1000% strain.

Furthermore, gaining insight into the extent of stiffening effects is also important, as it offers us opportunities to choose the most suitable material for applications. Thus, the dσ/ds curves at different strain levels describe the change in modulus with the strain and allow evaluation of the stiffening range of the materials (Figure 2c). Accordingly, the stiffening range of material ***mix-co*****-P** and **VHB** started at 220 and 200%, respectively, defined as the starting point of the stiffening range (*s*_0_). Next, the elastic modulus at *s*_0_, which was supposed to be the minimum elastic modulus, *Y*_min_, was determined. From observation, both materials had stiffening effects from *s*_0_ until strain at break. Thus, all materials’ elastic modulus at the end of the stiffening effect, which is the maximum elastic modulus *Y*_max_, were obtained accordingly (Table 1). ***mix*****-*****co*****-P** resembles **VHB** in many mechanical properties, such as similar *Y*_min_ (34 vs **VHB**’s 38 kPa) and similar *s*_0_ (220% vs **VHB**’s 200%). However, ***mix*****-*****co*****-P** stiffened more dramatically than **VHB**, showing higher *Y*_max_ (199 vs **VHB**’s 103 kPa) and higher *Y*_max_/*Y*_min_ ratio (5.9 vs **VHB**’s 2.7) (Figure 2c and Table 1). In addition to ***mix*****-*****co*****-P**’s superior stiffening effect over **VHB**, ***mix*****-*****co*****-P** also had longer strain at break (830% vs **VHB**’s 757%), suggesting that ***mix*****-*****co*****-P** may be a more advantageous material for DEG than **VHB** in terms of mechanical properties.

***mix-co*****-P** was modified by a thiol–ene reaction with thiopropionitrile, whereby some of the double bonds of the polynorbornene backbone were converted to more flexible single bonds. Accordingly, modified polymer **mix-*****co*****-CN** is expected to have a decreased elastic modulus compared to ***mix-co*****-P**. On the other hand, the density of dipoles in the modified polymer is increased, which should lead to an increased elastic modulus after the chemical modification. The overall result depends on the trade-off between the two effects. For instance, in our previous work, a homo bottlebrush polymer ***synthesized starting from mix***-**M** and modified by the same polar thiol gave a material with an elevated elastic modulus.^41^ This is probably due to the homo bottlebrush polymer’s high density of the side chains that hampered the flexibility of the backbone to a certain degree. Therefore, the dipolar interaction was dominated by a material with an increased elastic modulus. However, due to the polar group modification, self-segregation of the polynorbornene in **mix-*****co*****-CN** may be less effective, leading to a softer material with a smaller *Y*_min_ (17 vs ***mix*****-*****co*****-P’s** 34 kPa) and extended strain at break (1553% vs ***mix*****-*****co*****-P’s** 830%) (Figure 2b). The sparse long side chains may account for this outcome since they boost the backbone’s flexibility. However, the strain stiffening effect weakened for ***mix*****-*****co*****-CN** after the polar group modification since the *Y*_max_/*Y*_min_ ratio reduced to 3.9 (vs ***mix*****-*****co*****-P’s** 5.9). Unlike the other materials, the end of the stiffening range of ***mix*****-*****co*****-CN** is not marked by its strain at break, but occurs at 1000% strain (Figure 2c). Therefore, the *Y*_max_ value of ***mix*****-*****co*****-CN** is the elastic modulus at 1000% strain. Besides, though the stiffening effect of ***mix*****-*****co*****-CN** did not improve after the functionalization, it is still better than **VHB**, as indicated by the higher *Y*_max_/*Y*_min_ ratio (3.9 vs **VHB**’s 2.7).

The stiffening effect plays a key role in multiple applications. Therefore, restoring the undermined stiffening capability of ***mix*****-*****co*****-CN** may still be worthwhile at some cost of other aspects like increased elastic modulus. Therefore, we chemically cross-linked the polymer ***mix*****-*****co*****-CN** by a bifunctional cross-linker (2,2′-(ethylenedioxy)diethanethiol) with a thiol group concentration of 1.1 mmol/g versus the mass of polymer ***mix*****-*****co*****-CN**, via thiol–ene reaction using 2,2-dimethoxy-2-phenylacetophenone (DMPA) as UV initiator (Scheme 1c). The new material was defined as ***mix*****-*****co*****-E**. To our delight, the stiffening ability of ***mix*****-*****co*****-E** was indeed achieved since the *Y*_max_/*Y*_min_ ratio increased to 6.1 (vs ***mix*****-*****co*****-CN**’s 3.9 and ***mix*****-*****co*****-P’s** 5.9). However, it did not come without any price, as *s*_max_ decreased to 289% and *Y*_min_ elevated to 147 kPa (Figure 2b,c and Table 1).

It should be noted that the change in the shape of the samples during the tensile test is not neglectable. Therefore, we also calculated the materials’ true stress from the engineering stress (Figure S7). Table S3 summarizes different mechanical parameters obtained by using the true stress. Although the mechanical performance of the materials in a biaxial stress–strain experiment might be more relevant to the materials’ deformation in a circular actuator, we provide evidence from the tensile test that it is important to consider the real stress for the particular mechanical setup being used.

**Figure 3:**
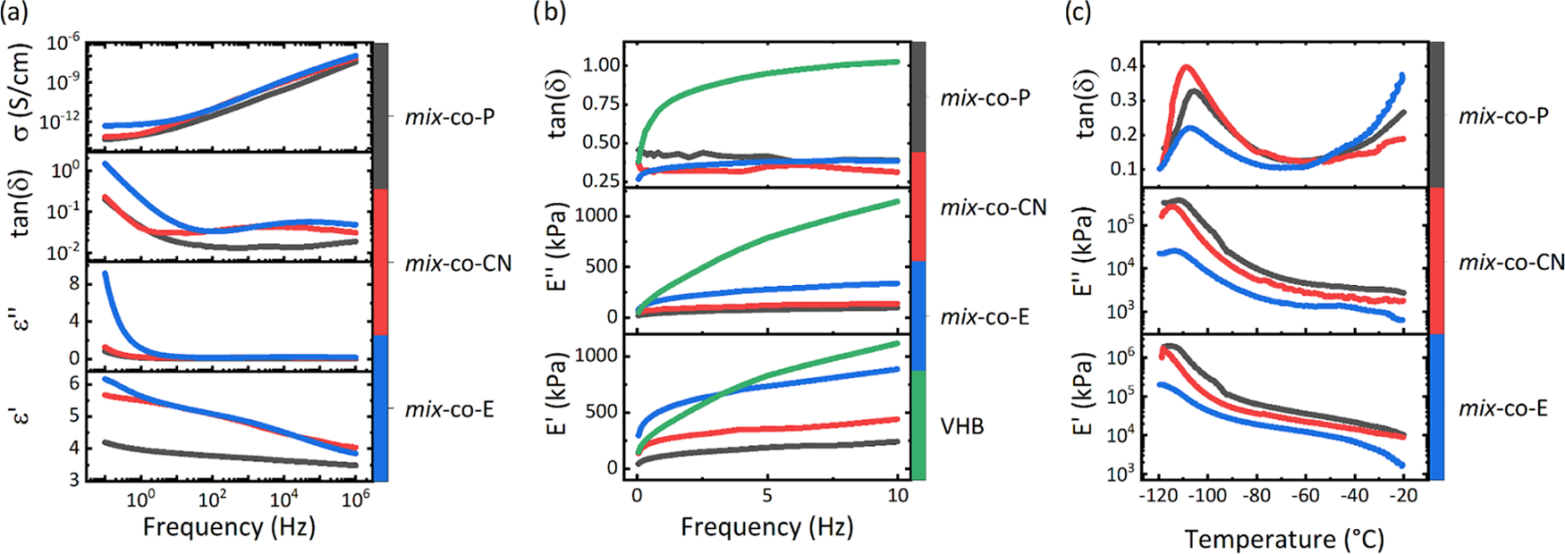
Dielectric permittivity (ε′), dielectric loss (ε″), loss factor (tan δ), and conductivity (σ) of the materials as a function of frequency at room temperature (a). The dynamic mechanical response of different materials at frequencies ranging from 0.05 to 10 Hz at 2% strain (b). The dynamic mechanical response of different materials at a frequency of 6 Hz at 2% strain in the temperature between −120 and −20 °C (c).

After the tensile test, we explored the materials’ dielectric properties (Figure 3a). The relative permittivity of ***mix*****-*****co*****-P** was 3.83 at 20 Hz. After the polar group modification, the dielectric permittivity increased to 5.23, which is higher than the VHB of 4.45–4.48 (1–10 Hz).^46−49^ Besides, tan δ (ε″/ε′ ratio) revealed that our materials can be operated at frequencies above 1 Hz where tan δ remains lower than 0.1. Additionally, the conductivity of all materials was below 10^–12^ S/cm, which is rather low and further confirms the good dielectric properties of our materials.

In addition, a dynamic mechanical analysis (DMA) was conducted (Figure 3b). For every material, three samples in stripe shapes were tested independently at room temperature at a strain of 2%. In the figure, the averaged parameters, including storage modulus (*E*′), loss moduli (*E*″), and mechanical loss factor (tan δ) within the frequency of 50 mHz and 10 Hz were presented. All materials showed storage modulus ranging from 160 to 750 kPa. Compared with VHB, our materials showed significantly lower mechanical loss factors. Additionally, VHB shows a stronger change in the elastic modulus with the frequency. Therefore, it can be foreseen that our materials exhibit superior properties for DEG applications due to the lower mechanical losses during operation. Mechanical losses heat the material and may cause an early dielectric breakdown.

Temperature-dependent DMA measurements were conducted to find the different transition temperatures in our materials (Figure 3c). Although the measurements at higher temperatures were noisy, the data from −120 to −20 °C was good. Since the interplay between the flexibility brought by newly formed single bonds and the dipolar interaction from increasing polar group density was uncertain, it is interesting to compare the *T*_g_ of ***mix*****-*****co*****-P** and ***mix*****-*****co*****-CN**. The measurement revealed a *T*_g_ for ***mix*****-*****co*****-P** at −105.6 °C, while the *T*_g_ of ***mix*****-*****co*****-CN** was −108.7 °C. The decreased *T*_g_ of ***mix*****-*****co*****-CN** complied with the result of mechanical characterization. Besides, the *T*_g_ of ***mix*****-*****co*****-E** was −106.9 °C, which slightly increased compared with ***mix*****-*****co*****-CN** as expected for a cross-linked material. However, it was still lower than that of the precursor ***mix*****-*****co*****-P**. Table S4 gives an overview of the transition temperatures observed in the different measurements.

**Figure 4:**
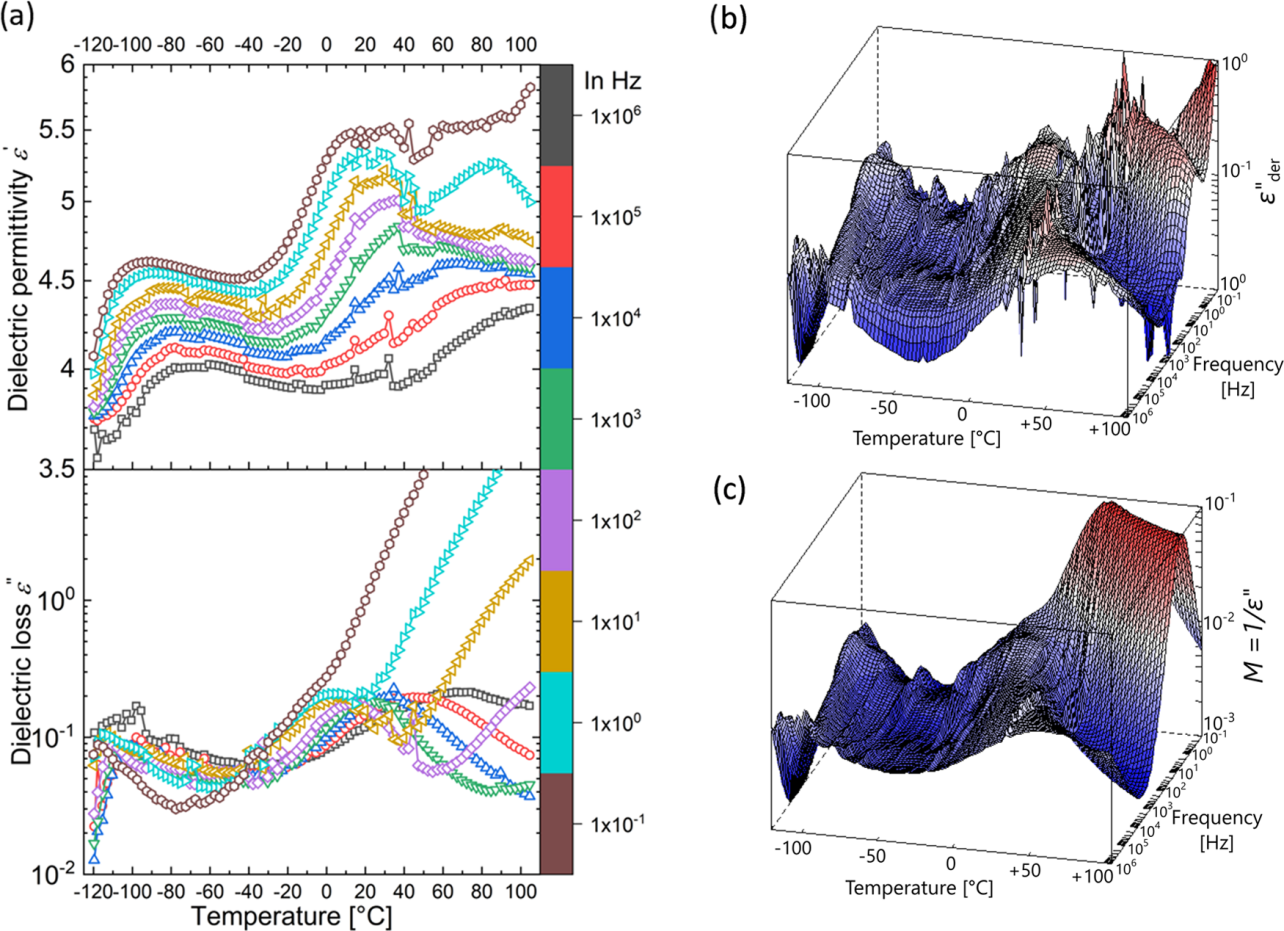
Real and imaginary parts of dielectric permittivity of ***mix*****-*****co*****-CN** as a function of temperature from −120 to 105 °C at selected frequencies from 10^–1^ to 10^6^ Hz (a). 3D plot of conduction-free ε″_der_ curves of a **mix-*****co*****-CN** cross-linked brush polymer as a function of frequency from 10^–1^ to 10^6^ Hz and temperatures from −120 to 105 °C (b). 3D plot of imaginary electric modulus, *M*″ of a ***mix*****-*****co*****-CN** brush polymer as a function of frequency from 10^–1^ to 10^6^ Hz and temperatures from −120 to 105 °C (c). It should be noted that ***mix*****-*****co*****-CN** makes free-standing films by physical cross-linking.

Measurement of the dielectric permittivity and losses at different temperatures allows us to observe the various relaxations and transitions occurring in the bottlebrush polymer elastomers. Figure 4a contains the isochronal plot of the real and imaginary parts of complex dielectric permittivity ε* commonly termed dielectric permittivity ε′ and dielectric loss ε″, respectively, of the polar bottlebrush copolymer **mix-*****co*****-CN**.

Permittivity curves reveal two frequency-dependent relaxations, i.e., processes whose curves shift to higher temperatures with increased frequency. These two processes are manifested in the loss plot as peaks in the same temperature ranges. The contribution from DC conductivity commonly observed at high temperatures and low frequencies in the form of a steep increase in permittivity or losses obscures the information from other relaxation processes occurring in these conditions. Subjecting the dielectric data to a derivative analysis makes it possible to separate the ohmic conduction.^50,51^ Commonly, a frequency derivative of permittivity, which is referred to as the conduction-free dielectric loss derivative ε″_der_ is used as shown in eq 1.
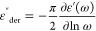
1A three-dimensional (3D) plot of the conduction-free loss of ***mix*-*****co*****-CN** is shown in Figure 4b, which reveals an additional frequency-dependent process at temperatures above 50 °C in addition to the two relaxations observed in Figure 4a at lower temperatures.

**Figure 5:**
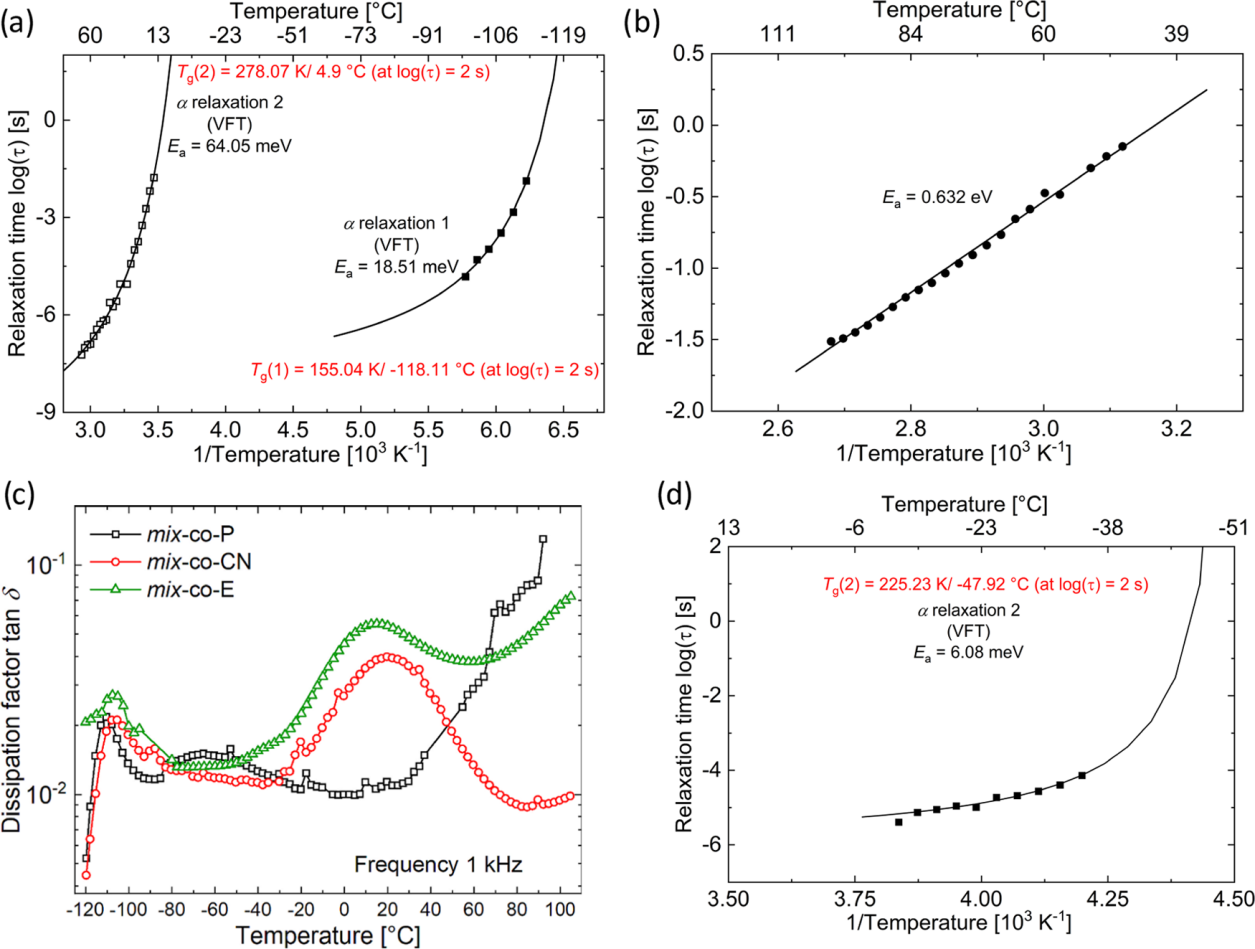
Arrhenius plot of relaxation processes showing a VFT fit for the ***mix*-*****co*****-CN** sample (a). Arrhenius plot of the high-temperature process shows the Arrhenius type of relaxation in the ***mix*-*****co*****-CN** sample (b). Dissipation factor tan δ for the three polymer brush samples as a function of temperature measured with a frequency of 1 kHz (c). Arrhenius plot showing the VFT fit for a ***mix*****-*****co*****-P** sample (d).

The Havriliak–Negami (HN) function was used to fit the relaxation peaks and to derive Arrhenius plots for each of the three processes. DCALC program developed by Wübbenhorst et al. was used for the fitting process and the plots are shown in Figure 5a,b.^50,51^ The two processes observed in Figure 4a show a nonlinear dependence of relaxation times with temperature in Figure 5a, which is characteristic of a Vogel–Fulcher–Tammann (VFT) relaxation.^52^ A VFT behavior denotes the dipolar relaxation occurring at the onset of segmental motions in amorphous polymers as the polymer is heated through its *T*_g_. This is the strongest relaxation; hence, it is referred to as the α relaxation. Since both relaxations show VFT behavior, we can conclude that the brush copolymer shows two *T*_g_’s, which is unexpected for a random copolymer. Therefore, the presence of a second *T*_g_ is an indirect proof for the self-segregation of the nonpolar PDMS brushes and the polar and less flexible polynorbornene backbone.

From an Arrhenius plot showing VFT behavior, one can calculate the dynamic *T*_g_ by extrapolating the curve to a relaxation time of 100 s (log τ = 2s).^53^ Starting from low to high temperature, two *T*_g_’s can be estimated for the brush copolymer, as shown in Figure 5a. While α_1_ relaxation yields a value of −118 °C, α_2_ relaxation shows a higher value around 5 °C. Concerning the structure, as mentioned earlier, a ***mix*-*****co*****-CN** sample is essentially a ***mix*-*****co*****-P** sample modified by a polar group (3-mercapto propionitrile) via a thiolene reaction. Hence, assigning the lower *T*_g_ to arise from the PDMS brushes is reasonable but slightly higher than regular PDMS with *T*_g_ = −127 °C (Figure 5a). The second *T*_g_ is likely due to polynorbornene aggregation. Also, the DMA analysis at different temperatures suggests the presence of two *T*_g_ values for the three synthesized materials. The first is at temperatures below −110 °C, while the second can be observed above −20 °C. Unfortunately, the DMA measurement is too noisy, so the maximum in the tan δ, which gives the value of the second *T*_g_ cannot be easily read.

It can be concluded that the current polar bottlebrush copolymers exhibit phase segregation, and therefore, two *T*_g_’s can be observed. Phase segregation introduces interfaces that lead to Maxwell–Wagner Interface (MWI) polarization, which occurs above *T*_g_ and can be observed by impedance spectroscopy.^54,55^ Looking at Figure 5b, which shows the Arrhenius plot of the high-temperature relaxation observed in Figure 4a, we can assign the Arrhenius type of relaxation behavior with a higher activation energy to MWI polarization. However, it is important to consider the contributions from electrode polarization, whose effects are observed at these elevated temperatures and low frequencies,^56^ leading to electrical conductivity relaxation (ECR).^57^ This typically arises due to an accumulation of space charges at the interface of the electrode and the dielectric sample. Plotting the complex electric modulus, M* = *M*′ + *jM*″, which is the inverse of complex dielectric permittivity (M* = 1/ε*) can help in this regard. Accordingly, in Figure 4c, the imaginary part of M* (*M*″ = ε″/(ε′^2^ + ε″^2^)) of a mix-*co*-CN sample is plotted as a function of temperature and frequency. The advantage of using electric modulus is that the effects of electrode polarization are suppressed at low frequencies and at high temperatures.^58,59^Figure 4c shows the 3D temperature and frequency plot of *M*″ of the sample under consideration. The strong presence of the high-temperature relaxation supports the idea that it arises from MWI interface polarization rather than from electrode polarization.

Relaxations in ***mix*****-*****co*****-P** and ***mix*****-*****co*****-E** samples were also studied (Figure S8), where the conduction-free loss ε″_der_ was plotted in 3D versus temperature and frequency. Both samples show a low *T*_g_ that can be assigned to the polymer brushes, as observed in ***mix*****-*****co*****-CN** (Figure 5a). While a ***mix*****-*****co*****-P** sample shows a second transition between −75 and 25 °C (Figure S8), a ***mix*****-*****co*****-E** sample shows a higher and broader second transition between −25 and 70 °C. For the latter sample, this can be labeled as the second *T*_g_ based on a similar assignment in the ***mix*****-*****co*****-CN** sample, where the transition is observed in the same temperature range. For the former sample, an HN fit was made, followed by the plotting of an Arrhenius curve (Figure 5d). From the plot, VFT behavior is observed, denoting a downshifted second *T*_g_ at −48 °C. Finally, both samples above their respective second *T*_g_ showed an MWI polarization starting from 50 °C that shifts to higher temperatures with increasing frequency, confirming the phase segregation occurring within these materials.

Figure 5c compares the dissipation loss factor tan δ (tan δ = ε″/ε′) of all three samples at a frequency of 1 kHz. As mentioned above, all three samples have a low-temperature *T*_g_ relaxation peak associated with the polymer brushes. While the ***mix*****-*****co*****-CN** and ***mix*****-*****co*****-E** samples show a second glass transition temperature of around 20 °C, the ***mix*****-*****co*****-P** sample has a lower *T*_g_ of −65 °C. Since the former two samples have polar nitrile groups, they can be expected to unfreeze at a higher temperature than ***mix*****-*****co*****-P**. Carefully looking at Figure 5c shows a shoulder between −40 and −20 °C in a ***mix*****-*****co*****-E** sample. Attempts to fit the relaxation peaks with an HN fit were not successful, which suggests the shoulder is likely an artifact. However, further measurements and analysis will help to elucidate the behavior.

**Figure 6:**
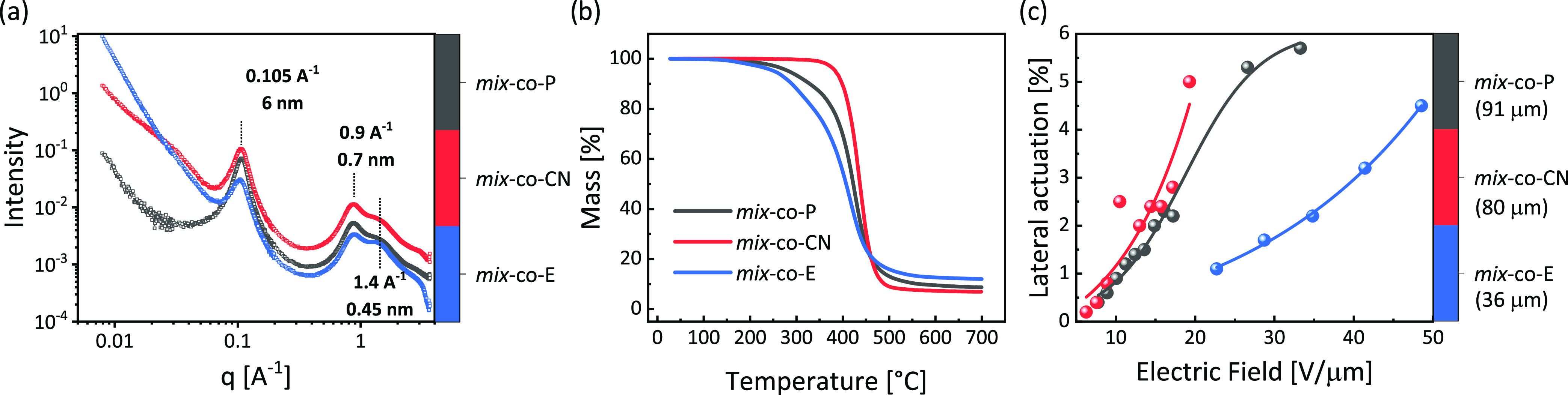
SAXS measurements of ***mix*****-*****co*****-P**, ***mix*****-*****co*****-CN**, and ***mix*****-*****co*****-E**. Thermogravimetric analysis (TGA) of the materials (a), and lateral actuation strains of thin films under different electric fields (***mix*****-*****co*****-P** prestrained by 100%, ***mix*****-*****co*****-E** prestrained by 150%) (b), and lateral actuation strain at different electric fields of the different materials (c).

Additionally, SAXS/wide-angle X-ray scattering (SAXS/WAXS) measurements were also conducted and revealed the presence of several peaks (Figure 6a). The first peak was observed at *q*_1_ = 0.105 Å^–1^, confirming phase segregation at a length scale of 6 nm. This peak corresponds to backbone–backbone space correlations among the bottlebrush chains.^60^ A WAXS higher order reflection at around *q*_2_ = 0.9 Å^–1^, characteristic of segmental electron density contrasts among chains, is indicative of the side chain-side chain packing at a length scale of 7 Å. A further WAXS Bragg reflection (third peak, *q*_3_) at these length scales is located at about 1.5 times *q*_2_, at a length of 4.5 Å. As there is no straightforward Bragg ratio corresponding to *q*_3_:*q*_2_ = 1.5, we tentatively suggest a rectangular packing with these two length scales (7 and 4.5 Å). More complex oblique columnar packing is also possible but would require additional reflections to resolve the angle, which are not visible and, therefore, cannot be proposed.

Thermogravimetric analysis (TGA) disclosed the thermal stability of the materials from RT to 700 °C under an inert atmosphere (Figure 6b). No significant loss was spotted up to 200 °C for all materials. Interestingly, ***mix*****-*****co*****-CN** exhibited a more stable feature with a loss peak above 350 °C, while ***mix*****-***co***-P** started to lose mass at 200 °C. ***Mix-co*****-P** has double bonds in the repeat unit that make this polymer susceptible to degradation. ***Mix-co*****-CN** was achieved by chemical modification of ***mix-co*****-P** with a polar thiol, whereby likely the most reactive double bonds were consumed, which may explain its higher thermal stability. Finally, ***mix-co*****-E** was obtained by cross-linking ***mix-co*****-CN** with a thiol. The lower thermal stability of ***mix-co*****-E** may be explained by the presence of some residual thiols or disulfide, which can generate radicals, lowering the degradation temperature.

After verifying the dielectric and mechanical properties, our materials were investigated as dielectrics in electromechanical tests. Circular actuators were constructed by fixing a thin membrane between two rigid circular frames and applying overlapping carbon black electrodes on both sides of the membrane. All films were free-standing, without additional pressure, unless otherwise noted concerning the prestraining percentage. We summarized the materials’ lateral actuation versus the electric field in Figure 6c. As we have pointed out, ideally, it is better to prestrain these elastomers at least until their s_0_ strain to get access to higher breakdown fields by entering the stiffening range and thus circumventing the EMI effect. However, some materials have quite low *Y*_max,_ values, as shown in Table 1. For instance, ***mix-co*****-P** exhibited a *Y*_max_ = 199 kPa and ***mix-co*****-CN** a *Y*_max_ = 68 kPa. Hence, as the membrane undergoes prestretching and thinning, the maximum force that the film can withstand diminishes. Consequently, even if some membranes survived prestretching, most still ruptured when manually applying the carbon black electrodes. Consequently, some materials can only be prestrained until a strain that is less than s_0_ without rupturing. Film of ***mix*****-***co***-P** was biaxially prestrained by 100% instead of 220% (Figure 6c). Nevertheless, the breakdown field of ***mix*****-*****co*****-P** still reached 30.7 V/μm, while it actuated laterally by 5.7%. Due to the chemical modification of ***mix*****-*****co*****-CN**, the elastic modulus was decreased and gave similar actuation at lower electric fields. Furthermore, the breakdown field of ***mix*****-*****co*****-CN** decreased as well, which is expected since the stiffening effect decreased. The strain stiffening effect was most pronounced in material ***mix*****-*****co*****-E**, which substantially improved the dielectric breakdown field. However, this material was rather stiff and had a low actuation. Table 2 summarizes the breakdown fields of ***mix*****-*****co*****-P**, ***mix*****-*****co*****-CN**, and ***mix*****-*****co*****-E** as 30.7, 33.9, and 62.4 V/μm, respectively. Figure S9 shows the cyclic actuation of ***mix*****-*****co*****-E** at 0.25 Hz, which has a small hysteresis in the five cycles and after that stabilizes. The low actuation strain, high dielectric breakdown field, increased dielectric permittivity, and good stretchability make ***mix*****-*****co*****-E** attractive as a dielectric elastomer in energy generators. The theoretical amount of energy harvested was calculated as 0.44 J/g for ***mix-co*****-P**, 0.98 J/g for ***mix-co*****-CN**, and 0.5 J/g for ***mix-co*****-E**, respectively (Table 2). More details regarding the calculations can be found in the SI. The amount of energy calculated is in the same range as VHB and thus in agreement with the literature.^40^

**Table 2:** Lateral Actuation Strain at the Maximum Electric Breakdown (*s*), Dielectric Breakdown (*E*_b,max_), and Original Film Thickness (*d*_0_)

entry	*s*^a^ [%]	*s*_areal_^b^ [%]	voltage^c^ [V]	*d*_0_^d^ [μm]	Δ*d*^e^ [μm]	*E*_b,max_^f^ [V/μm]	ε′^h^	Δ*E*_dens_ [J/g]
***mix-co*****-P**^g^	5.7	11.7	2500	91	9.5	30.7	3.83	0.44
***mix-co*****-CN**	4.2	8.6	500	16	1.3	33.9	5.23	0.98
***mix*****-*****co*****-E**	5	10.2	600	36	0.4	62.4	5.24	0.5

a Lateral strain at the maximum electric breakdown.

b Areal strain at the maximum electric breakdown.

c Voltage at the maximum electric breakdown.

d Original thickness of the thin film.

e Change in thickness Δ*d* = *d*_0_ – *d*, considering the corresponding thickness *d*: *d* = *d*_0_/(*s* + 1)^2^.

f Exact breakdown fields of the thin film given by *E*_b,act_ = [Voltage]/*d*.

g Prestrained by 100%.

h Taken at 20 Hz.

## 4 Conclusions

A bottlebrush polymer was synthesized by copolymerizing a macromonomer of norbornene ester modified with a PDMS chain and 5-norbornene-2-carbonitrile via ROMP. ^1^H NMR investigations show that the two monomers are randomly incorporated into the polymer chain. The copolymer formed has double bonds in its backbone, facilitating a thiol–ene reaction with thiopropionitrile to enhance the dielectric permittivity and enable cross-linking with a bifunctional thiol. The as-prepared bottlebrush polymer and the one resulting from postpolymerization modification with thiopropionitrile form free-standing films with good elastic properties after solvent casting. Bottlebrush polymers entangle much less than their common counterparts. Since both candidates discussed here are random copolymers, it was questionable what physical interactions keep these two polymers in shape. DMA and impedance spectroscopy analysis reveal the presence of two *T*_g_ in both cases, which is surprising for random copolymers and asks for an explanation. Additionally, the presence of a third relaxation process at higher temperatures in impedance spectroscopy suggests the presence of interfaces, giving rise to interfacial polarization. Furthermore, SAXS measurements confirmed the presence of segregation, with PDMS domains with a *T*_g_ at below −100 °C in both cases and polynorbornene backbone domains with *T*_g_’s at −48 °C (for ***mix*****-*****co*****-P**) and 4.9 °C (for ***mix*****-*****co*****-CN**), respectively. It is reasonable to assume that this domain formation is triggered by the polynorbornene backbone’s poor flexibility and the many nitrile groups on the backbone. Moreover, the remaining double bonds of the bottlebrush polymer modified with polar groups allow for chemical cross-linking via a thiolene reaction with a bifunctional thiol, thus further optimizing the mechanical properties. The chemically cross-linked bottlebrush polymer exhibits a dielectric permittivity of 5.24 and strain stiffening, which may prevent the dielectric from EMI. Additionally, the material exhibits large strain at break of about 300% and lower mechanical losses than VHB, one of the most explored dielectrics in generators. Due to the increased elastic modulus, the material is poorly actuated but withstands a high breakdown field of up to 62.4 V/μm. Therefore, this material exhibits attractive properties for applications as a dielectric in dielectric elastomer generators, which should be explored in the future.

## Supporting Information Available

NMR spectra; GPC curves; true stress–strain curves; 3D ε″_der_ plot as a function of temperature; electromechanical measurements; photos of the elastomer; and calculations of the maximum theoretical energy that can be harvested from a DEG operated in uniaxial stretching mode (PDF)
