# Supplementary material for: Synthesis of Bottlebrush Polymers with Spontaneous Self-Assembly for Dielectric Generators
Source: ACS Appl Polym Mater. 2024 Apr 22;6(9):4999–5010. doi: 10.1021/acsapm.3c03053 (PMC11091855; doi:10.1021/acsapm.3c03053)
Supplement: Supplementary file 1 — ap3c03053_si_001.pdf [file ap3c03053_si_001.pdf]

Supporting Information

Synthesis of bottlebrush polymers with spontaneous self-assembly for dielectric generators

Yeerlan Adeli,[a,b] *Thulasinath Raman Venkatesan,* [a] *Raffaele Mezzenga,*[c] *Frank A. Nüesch,*[a,b] *Dorina M. Opris*[a,d]*

[a]Laboratory for Functional Polymers, Swiss Federal Laboratories for Materials Science and Technology Empa, Ueberlandstr. 129, CH-8600, Dübendorf, Switzerland

[b]Institute of Chemical Sciences and Engineering, Ecole Polytechnique Federale de Lausanne, EPFL, Station 6, CH-1015 Lausanne, Switzerland

[c]Department of Health Sciences and Technology, ETH Zürich, Laboratory of Food and Soft Materials, Schmelzbergstrasse 9, 8092 Zürich, Switzerland

[d]Department of Materials, ETH Zurich, Vladimir-Prelog-Weg 5, 8093 Zurich, Switzerland

E-mail: dorina.opris@empa.ch

KEYWORDS: dielectrics, bottlebrush polymers, electrically-responsive elastomers, ROMP, self-segregated copolymers

EXPERIMENTAL SECTION

^{1}H and ^{13}C NMR spectra were recorded with a Bruker AVANCE 400 NMR spectrometer using a 5 mm BBO ProdigyTM CryoProbe at 400.18 and 100.63 MHz, respectively. Chemical shifts (δ) in ppm are calibrated to the residual solvent peak ($CDCl_3$: δ = 7.26; 77.16).

Gel permeation chromatograms were recorded using an Agilent 1100 Series HPLC (columns: serial coupled PSS SDV 5 m, 100 Å and PSS SDV 5 m, 1000 Å, detector: DAD, 235 and 360 nm; refractive index), with THF as the mobile phase. PDMS standards were used for calibration and toluene as an internal standard.

Thermogravimetric analysis (TGA) was conducted with a PerkinElmer TGA7 in either a nitrogen or helium atmosphere by heating the sample from 30 to 900 °C at a speed of 20 °C/min.

The tensile tests were performed using a Zwick Z010 tensile test machine with a cross-head speed of 50 mm min^{-1}. Tensile test specimens with a gauge width of 2 mm and a gauge length of 18 mm were prepared by die-cutting. The strain was determined using a longitudinal strain extensometer. The curves were averaged from different independent experiments. The Young's modulus at 10% strain was determined from the slope of the stress−strain curve using a linear fit to the data points from 0 to 10% strain. While for the Young's moduli at x% other than 10%, a linear fit to the data points from (x-5)% to (x+5)% strain, respectively, was used.

Dynamic mechanical analysis was carried out on a RSA 3 DMA from TA Instruments. Stripes of 10 mm × 20 mm were measured under a dynamic load of 2 g, at 2% strain in the frequency range of 6 Hz at 25 °C. The mechanical loss factor tan (δ) is given as the fraction of imaginary and real storage modulus at 2% strain.

Dielectric measurements were performed in the frequency range from 1 to 10^6 Hz using a Novocontrol Alpha-A frequency analyzer. The VRMS (root mean square voltage) of the probing ac electric signal applied to the samples was 1 V. The relative permittivity ε' was determined from the capacitance $C = \varepsilon'\varepsilon_0 A/d$, where A is the electrode area, d is the thickness of the film, and ε_0 is the vacuum permittivity. The thickness of the film was measured by a micrometer gauge with an uncertainty of ±5 μm. The samples were prepared by placing the film between two stainless steel discs with a diameter of 15 mm. Before measurement, the samples were annealed at 80 °C in a vacuum oven for 24 hours.

Electromechanical tests were performed using circular membrane actuators at ambient temperature and humidity. Before crosslinking the bottlebrush polymer, a substrate was made by casting PVA with a thickness of 400 μm on a glass substrate. After PVA solidified in 1 hour, the mixture of bottlebrush polymer was cast on the PVA substrate and cross-linked. Then the films together with the PVA substrate were fixed between two circular rigid frames with an inner diameter of 25 mm. To remove the PVA substrate, the fixed film and circular rigid frames were put into de-ionized warm water with 60 °C. The water was constantly changed every hour. After about 6 h, the fixed film and circular rigid frames were put into vacuum oven with 60 °C to remove residual solvent and water. Circular electrodes (8 mm diameter) of carbon black powder was applied to each side of the film. A FUG HCL- 35-12,500 high voltage source served as a power supply for actuator tests. We gradually increase the voltage by 100 V and up to 2000 V. The actuation strain was measured optically as the extension of the diameter of the electrode area via a digital camera, using an edge detection tool of a LabView program to detect the boundary between the black electrode area and the transparent film.

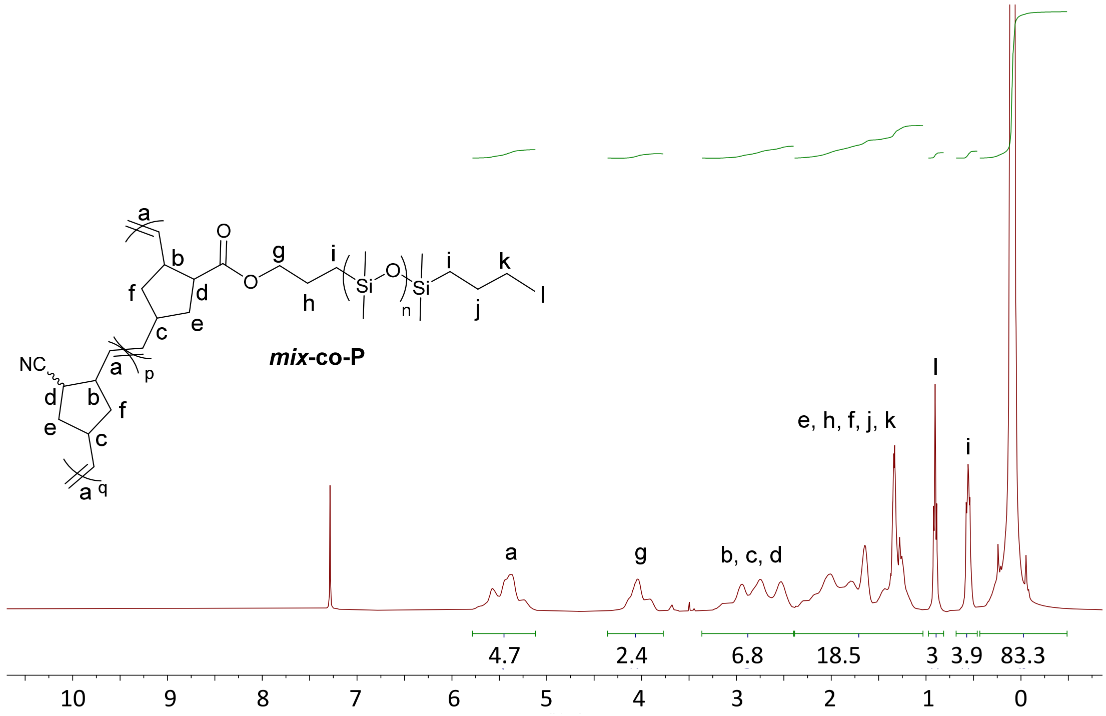

Figure S1. ^{1}H NMR spectrum of ***mix*-co-P** in $CDCl_3$. The ratio of p : q was calculated as 2 : (a-2) = 2 : 2.72.

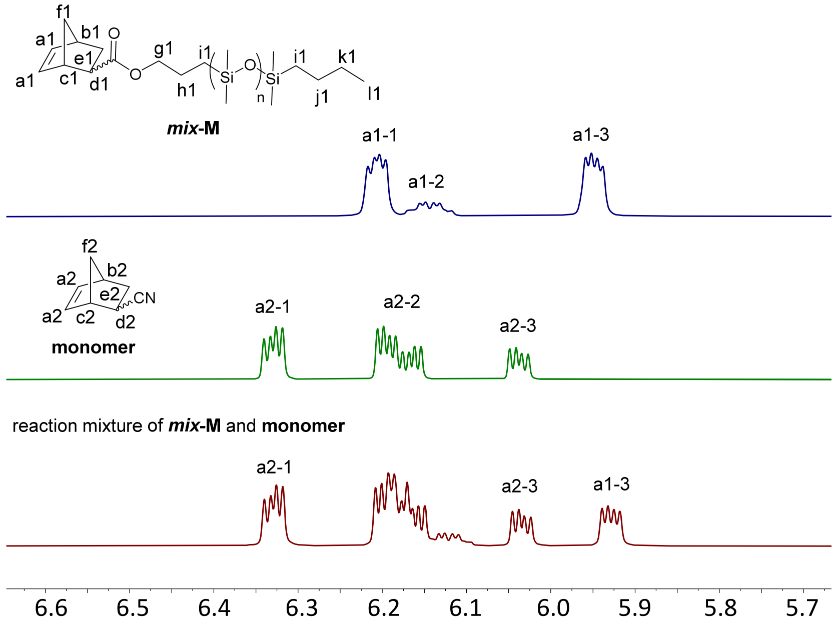

Figure S2. ^{1}H NMR spectra of macromonomer ***mix*-M** (top), **monomer** (middle), and polymerization mixture of ***mix*-M** and ***mix*-co-P** (bottom). The conversions of ***mix*-M** at x min was decided by 1 - "a1-3 (at x min)"/ "a1-3 (at 0 min)". As for the conversions of monomer, first the average integration of "a2-1" and "a2-3", "a2-ave" was calculated. Then conversions of monomer at x min = 1 - " a2-ave (at x min)"/ " a2-ave (at 0 min)". The results are summarized in **Table S2**.

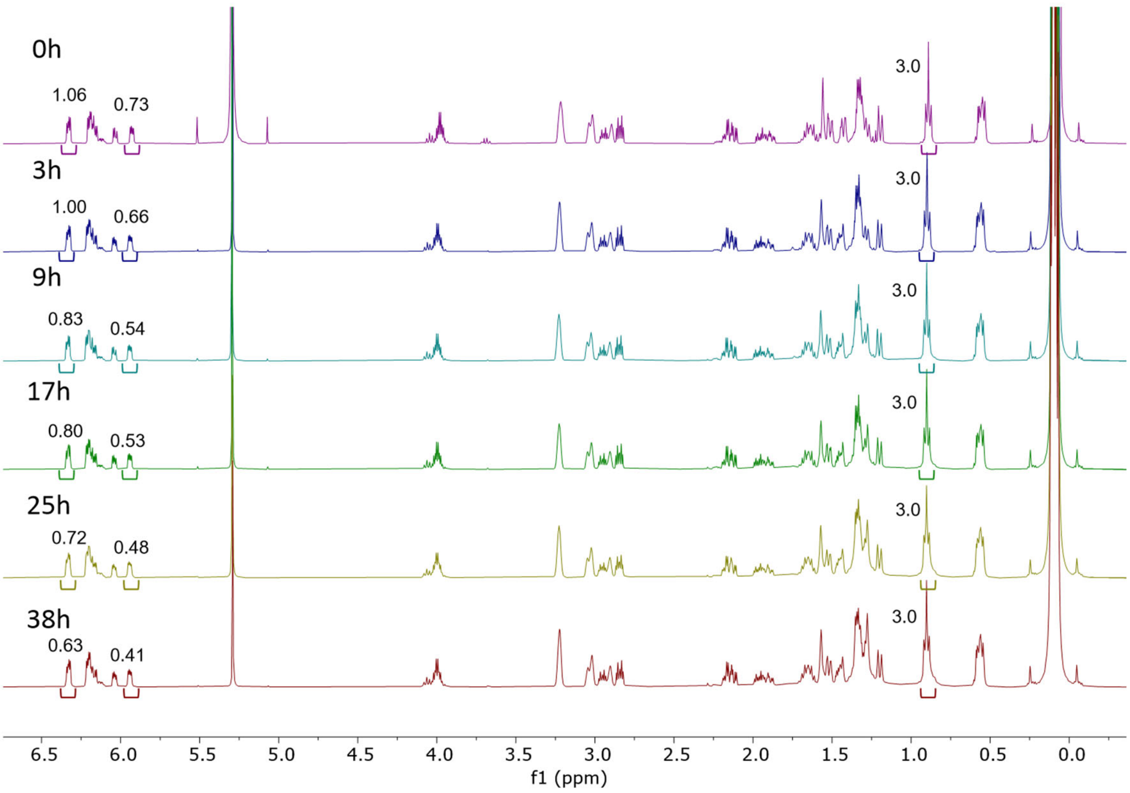

Figure S3. Selective ^{1}H NMR spectra of the polymerization mixture solution over time.

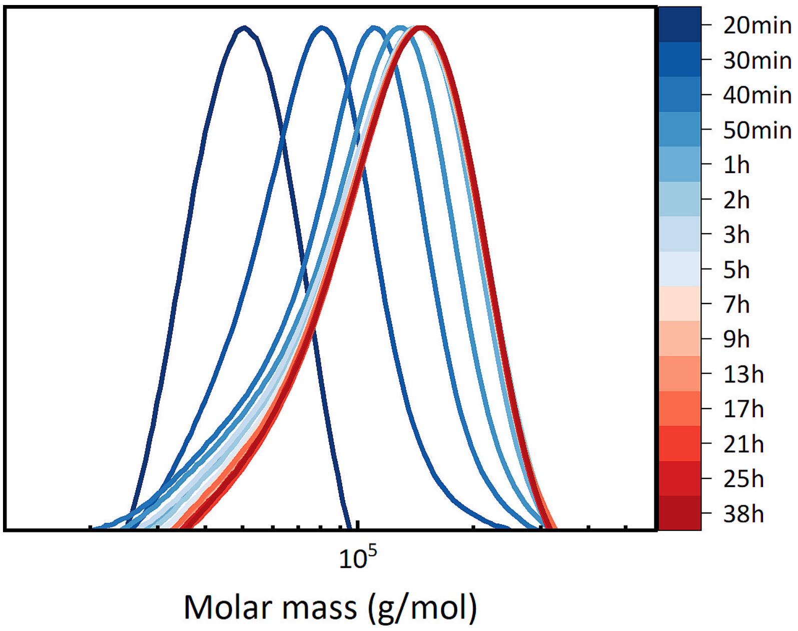

Figure S4. GPC curves of the polymerization mixture solution at different reaction times using THF and polydimethylsiloxane standards.

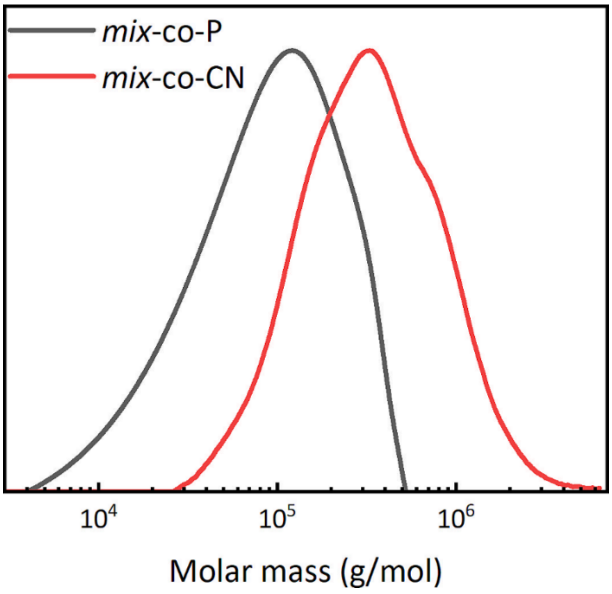

Figure S5. GPC curves of ***mix*-co-P** and ***mix*-co-CN** using THF and polydimethylsiloxane standards.

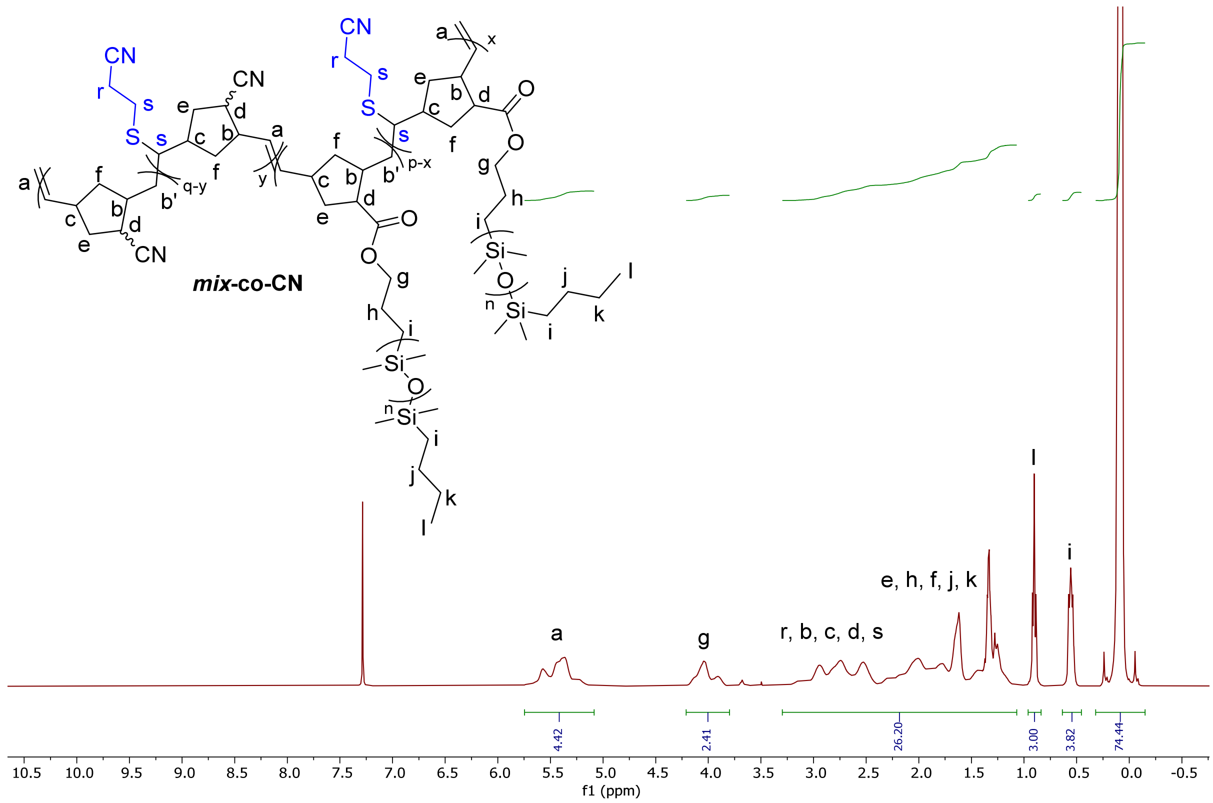

Figure S6. ^{1}H NMR spectrum of ***mix*-co-CN** in $CDCl_3$.

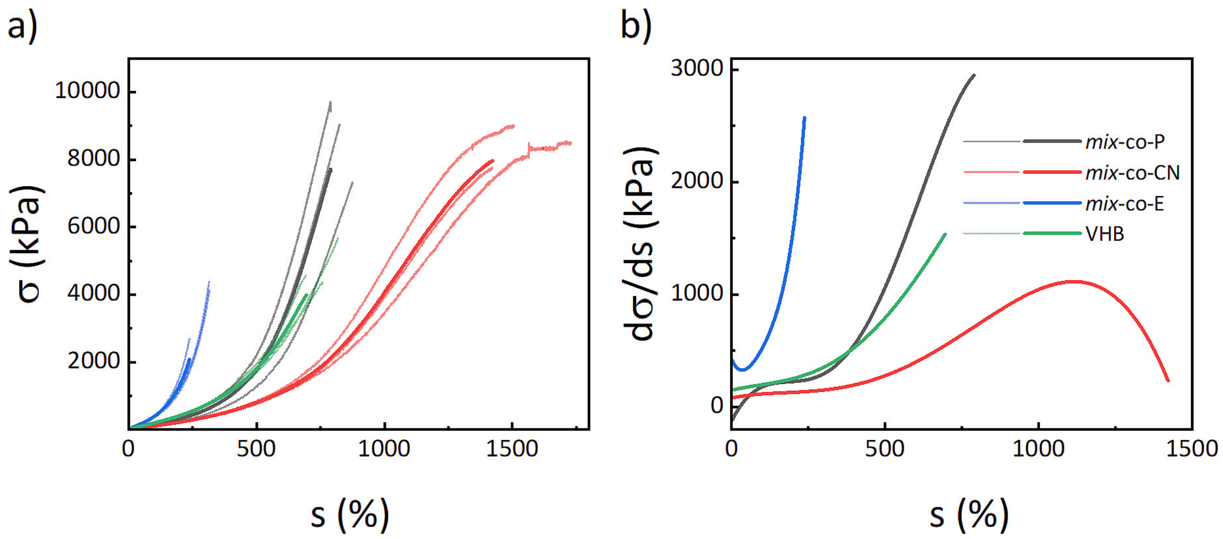

Figure S7. True stress-strain curves for materials ***mix-co*-P**, ***mix-co*-CN**, ***mix-co*-E**, and **VHB**. The light curves are the results from 3 independent measurements of the same material, while the bold curves are the averaged curves of the same material (a) and the curves of dσ/ds from true stress at different strains (b).

The true stress σ(true) was calculated from engineering stress σ(eng) while s is the extension ratio by this equation:

$$\sigma(\text{true}) = \sigma(\text{true}) * \ (1 + \text{s})$$

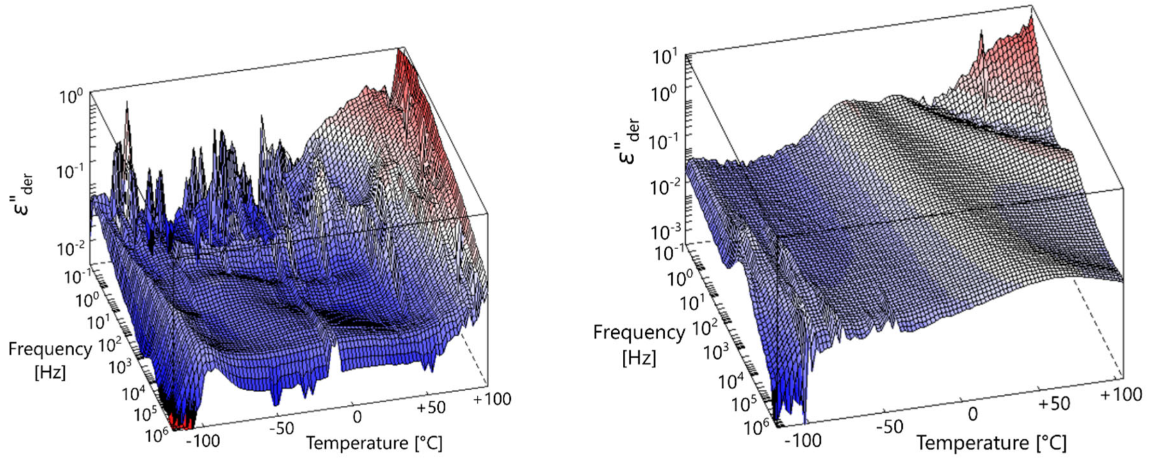

Figure S8. 3D ε''_{der} plot of a mix-co-P sample as a function of temperature (from -120 °C to 90 °C) and frequency (left). 3D ε''_{der} plot of a mix-co-E sample as a function of temperature and frequency (right).

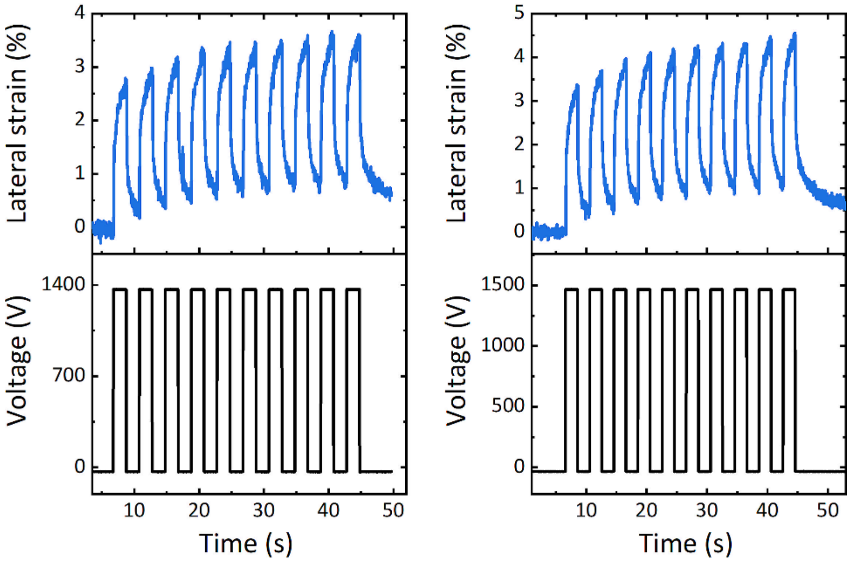

Figure S9. Electromechanical measurements at 0.25 Hz of films of ***mix*-co-E** pre-strain by 150% at an electric field of 41.4 V μm^{-1} (film thickness of 36.0 μm) (left) and 45.5 V μm^{-1} (film thickness of 36.0 μm) (right).

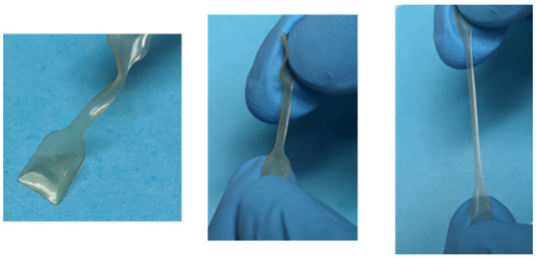

Figure S10. All materials were elastic, transparent with a slight yellowish colour.

Maximum theoretical energy that can be harvested from a DEG operated in uniaxial stretching mode

For DEG subjected to mechanical stretching, dielectric breakdown field E_b and electromechanical instability (EMI) are the main boundary conditions that dictate the performance.

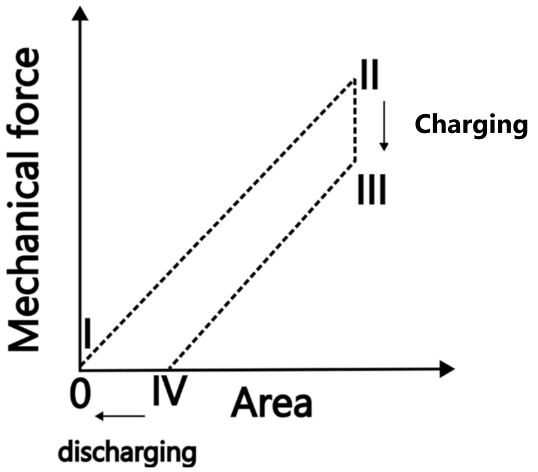

Figure S11. Proposed energy harvesting scheme during the operation of a DEG.

A dielectric material with thickness d_0 is covered with a compliant electrode of surface area A_0. In the first step, a uniaxial force is applied to the dielectric, whereby it is stretched from state I to II. Since we assume the incompressibility of the DEG, stretching leads to a decrease in the thickness and a corresponding increase in the surface area of the electrode. At this stage, the DEG is charged by switching on the voltage. The maximum electric field that can be applied to the dielectric is limited by its E_b. The deposition of charges on the electrode reduces the tension in the elastomer, resulting in a decrease in the mechanical force on the device (step III). At this stage, the voltage is disconnected, and the DEG is allowed to relax to its equilibrium position (IV) where the mechanical stress exerted by the elastomer balances the electrical stress (Maxwell pressure) induced by the electrode charges. Since the charges on the electrodes are constant and the surface area of the electrode is decreased, the voltage on the electrode increases during this step. Finally, the DEG is discharged and brought back to its original state to harvest the electrical energy.

The electrical energy ΔE that can be harvested from the DEG can be approximated by taking the difference between the energy stored in the capacitor in state IV and III, i.e. E_{IV} and E_{III}, respectively:

$$\Delta E = E_{IV} - E_{III};$$

(1)

$$E_{III} = \frac{1}{2} C_{III} {V_{III}}^2;\ E_{IV} = \frac{1}{2} C_{IV} {V_{IV}}^2, \tag{2}$$

where *C* is the capacitance, *V* is the voltage between the two electrodes. Since the DEG is incompressible, the overall volume remains constant, hence

$$A_0 d_0 = A_{III} d_{III} = A_{IV} d_{IV}. \tag{3}$$

The capacitance at stage III and IV can be expressed as:

$$C_{III} = \frac{A_{III}\, \varepsilon_0\, \varepsilon'}{d_{III}} = \frac{A_0\, d_0 \varepsilon_0\, \varepsilon'}{{d_{III}}^2} \text{ and } C_{IV} = \frac{A_0\, d_0\, \varepsilon_0\, \varepsilon'}{{d_{IV}}^2}. \tag{4}$$

Where ε' is the relative permittivity and ε_0 is the permittivity of vacuum. Inserting equations (2) and (4) in equation (1) gives:

$$\Delta E = \frac{1}{2} A_0\, d_0 \varepsilon_0 \varepsilon \left(\frac{{V_{IV}}^2}{{d_{IV}}^2} - \frac{{V_{III}}^2}{{d_{III}}^2}\right). \tag{5}$$

As charge q is constant at steps III and IV

$$C_{IV} V_{IV} = C_{III} V_{III} = q. \tag{6}$$

From equation (4) and (6) we have:

$$V_{IV} = \frac{C_{III} V_{III}}{C_{IV}} = \frac{{d_{IV}}^2\, V_{III}}{{d_{III}}^2}. \tag{7}$$

Substituting equation (7) in (5):

$$\Delta E = \frac{1}{2} \frac{A_0\, d_0 \varepsilon_0 \varepsilon\, {V_{III}}^2}{{d_{III}}^2} \left(\frac{{d_{IV}}^2}{{d_{III}}^2} - 1\right). \tag{8}$$

We set the boundary condition that the maximum charge q deposited during step II to step III on the electrodes corresponds to the voltage required to reach the breakdown field E_b. Hence:

$$V_{III} = E_b d_{III} \tag{9}$$

Substituting equation (9) in (8) we have:

$$\Delta E = \frac{1}{2} A_0\, d_0 \varepsilon_0 \varepsilon\, {E_b}^2 \left(\frac{{d_{IV}}^2}{{d_{III}}^2} - 1\right) \tag{10}$$

and assuming a density of $\rho = 10^6\ {}^{g}/_{m^3}$, the change in energy density can be described as:

$$\Delta E_{dens} = \frac{\Delta E}{Mass\ of\ actuator} = \frac{\Delta E}{A_0\, d_0\, \rho} = \frac{1}{2} \frac{\varepsilon_0 \varepsilon\, {E_b}^2}{10^6} \left(\frac{{d_{IV}}^2}{{d_{III}}^2} - 1\right) \left[\frac{J}{g}\right]. \tag{11}$$

It can be shown that ΔE_{dens} grows monotonously with decreasing d_{III}, i.e., higher stretches. As an approximation of the maximum gain of electrical energy, we can set:

$$d_{III} = \frac{d_0}{(S_{max}+1)^2}, \tag{12}$$

where s_{max} is the maximum strain that can be applied to the DEG.

Substituting equation (9) in (7) gives:

$$V_{IV} = \frac{E_b\, d_{IV}^{\ 2}}{d_{III}^{\ 2}}. \qquad (13)$$

In equilibrium at state IV, the elastic force on the sample is equal to the electrostatic force (Maxwell pressure). Hence,

$$\varepsilon\, \varepsilon_0 \left(\frac{V_{IV}}{d_{IV}}\right)^2 = Y_{IV} s_{IV}, \qquad (14)$$

where Y_{IV}, is the Young's modulus at state IV.

and from equation 12 we have:

$$d_{IV} = \frac{d_0}{(s_{IV}+1)^2}\ ;\ s_{IV} = \sqrt{\frac{d_0}{d_{IV}}} - 1. \qquad (15)$$

Rearranging equation (14) we get:

$$V_{IV}^{\ 2} = \frac{Y_{IV}\, s_{IV}}{\varepsilon\, \varepsilon_0}\, d_{IV}^{\ 2}. \qquad (16)$$

Equating (13) and (16) we have

$$d_{IV}^{\ 2} = \frac{Y_{IV}\left(\sqrt{\frac{d_0}{d_{IV}}}-1\right) d_{III}^{\ 2}}{\varepsilon_0 \varepsilon\, E_b^{\ 2}}. \qquad (17)$$

From equations (12) and (17), one can determine d_{III} and d_{IV}. Substituting these values and other known quantities in equation (11), we can calculate the theoretical maximum energy density ΔE_{dens} of the DEGs. To estimate the Young's modulus Y_{IV} of polymers mix-co-P, mix-co-CN, and mix-co-E, we used the corresponding true stress-strain curves from the tensile test (Figure S7). The results from this analysis are displayed in Table S5.

Table S1. Conversion of 5-norbornene-2-carbonitrile and ***mix*-M** over time.

Time (min)	5-norbornene-2-car-bonitrile (%)	*mix*-M (%)
0	0.0	0.0
60	0.9	0.0
120	3.7	2.9
180	7.3	5.7
300	12.8	12.9
420	17.4	17.1
540	23.4	22.9
780	24.8	24.3
1020	25.7	24.3
1260	35.8	35.7
1500	33.0	31.4
2280	40.8	40.0

Table S2. The molar masses and molar masses distributions result from GPC characterization.

**Entry**	**M_n [kDa]**	**M_w [Da]**	**PDI**
***mix*-co-P**	136.4	257.6	1.89
***mix*-co-CN**	209.3	453.1	2.16

Table S3. Mechanical properties of ***mix-co*-P**, ***mix-co*-CN**, ***mix-co*-E**, and VHB from true stress.

Entry	s_0 [%][a]	s_e [%][b]	Y_{min} [kPa][c]	Y_{max} [kPa][d]	Y_{max} / Y_{min}	s_{max} [%][e]
***mix-co*-P**[f]	27	s_{max}	139±21	2714±570	19.5	830±43
***mix-co*-CN**	0	1116	168±7	1105±149	6.6	1553±159
***mix-co*-E**	0	s_{max}	428±31	4397±698	10.3	289±45
**VHB**	0	s_{max}	273±3	1440±340	5.3	757±62

[a]The strain of the starting point of the stiffening range. [b]The strain of the ending point of the stiffening range. [c]Elastic modulus at s_0. [d]Elastic modulus at s_e. [e]Average strain at break of three samples.[f]Elastic modulus at 10% is 188±31 kPa.

Table S4. Transition temperatures obtained from Impedance and DMA measurements.

Sample		Impedance			DMA
		HN-fitting [°C]	*tan δ* at 5 Hz [°C]	*tan δ* at 1kHz [°C]	*tan δ* at 6 Hz [°C]
	T_g 1	-	-115.2	-110.3	-105 °C
**mix-co-P**	T_g 2	-47.9	-	-65.3	-
	T_g 1	-118.0	-112.8	-105.4	-108.7
**mix-co-CN**	T_g 2	5.0	2.2	19.7	-
	T_g 1	-	-112.8	-105.0	-106.9
**mix-co-E**	T_g 2	-	-5.24	15.0	-

Table S5. Calculated maximum electrical energy density ΔE_{dens} using equation (11). Initial thickness d_0, breakdown field E_b, relative permittivity ε, maximum tensile strain s_{max} and Young's modulus Y_{IV} were taken from experimental data.

	mix-*co*-P	mix-*co*-CN	mix-*co*-E
d_0 (µm)	91	16	36
E_b (MV/m)	30.7	33.9	62.4
ε	3.83	5.23	5.24
s_{max} (%)	830	1553	289
d_{III}(µm)	1.05	0.059	2.38
d_{IV}(µm)	5.61	0.36	6.07
s_{IV} (%)	302%	567	144
Y_{IV} (kPa)	300	355	818
ΔE_{dens} (J/g)	0.44	0.98	0.50
